# Suppression of circ_0091761 ameliorates acute myocardial infarction-induced endothelial injury through regulation of miR-1278

**DOI:** 10.1186/s41065-025-00519-z

**Published:** 2025-08-21

**Authors:** Bin Zhou, Haitao Wang, Kun Zhang, Jin Xie, Cuicui Yuan, Jia Jin, Jian Zhang, Meng Ma, Zhengnan Zhang

**Affiliations:** 1 Department of Emergency, Wuhan Third Hospital, Tongren Hospital of WuHan University, Hubei, 430060 China; 2 Department of Cardiology and Emergency Department, The 970th Hospital of the Joint Logistics Support Force of The Chinese People’s Liberation Army, Yantai, 264002 China; 3 Department of Critical Care Medicine, Zhangqiu District People’s Hospital, Jinan, 250200 China; 4 Department of Emergency, Zhangqiu District People’s Hospital, Jinan, 250200 China; 5 Department of Cardiology, Zhangqiu District People’s Hospital, Jinan, 250200 China; 6 Department of Pharmacy Zhangqiu District People’s Hospital, Jinan, 250200 China; 7 Cardiovascular Department, Geriatric Hospital Affiliated to Wuhan University of Science and Technology, No. 2, West Huangjiahu Road, Qingling Street, Hongshan District, Wuhan, Hubei Province 430065 China

**Keywords:** Acute myocardial infarction, circ_0091761, miR-1278, Endothelial injury

## Abstract

**Objective:**

The main objective of the article was to explore the role of circ_0091761 in acute myocardial infarction (AMI)-induced endothelial injury.

**Methods:**

A cellular model of AMI was constructed by hypoxia-induced HUVECs. miRNAs that may interact with circ_0091761 were recognized by the ENCORI and circular RNA Interactom databases. RT-qPCR was performed to analyze the levels of circ_0091761 in AMI patients as well as miR-1278, ICAM1, and VCAM1 in hypoxia-induced HUVECs. Flow cytometry was used to detect apoptosis. ROS kit and LDH kit were used to detect the levels of ROS and LDH, respectively. Dual-luciferase reporter assay, RIP assay, and RNA pull-down assay were performed to validate their interaction between circ_0091761 and miR-1278.

**Results:**

circ_0091761 was elevated in AMI patients compared to healthy controls. Silencing circ_0091761 reduced apoptosis, ICAM1, and VCAM1 levels, as well as ROS and LDH. circ_0091761 could interact with miR-1278 and negatively regulate miR-1278 expression in hypoxia-induced HUVECs. At the same time, inhibiting miR-1278 reverses the protective effect of transfected si-circ_0091761 on HUVECs.

**Conclusion:**

Down-regulation of circ_0091761 ameliorates AMI-induced endothelial injury by targeting miR-1278.

**Clinical trial number:**

Not applicable.

## Introduction

Acute myocardial infarction (AMI) is a prevalent cardiovascular disorder in clinic that causes high morbidity and mortality around the world [1, 2]. AMI attribute to primary reductions in coronary blood flow, which is caused by platelet attachment, release of humoral mediators, and endothelial injury at sites of coronary artery stenosis [3]. The function of endothelial can be altered by tissue ischemia induced by infarction, which has a key role in tissue adaption to injury [4].

Circular RNA (circRNA) is a class of non-coding RNA (ncRNA), which is a class of RNA with a closed loop structure connected in reverse through the 3’ and 5’ end [5, 6]. circRNA is stable, sensitive and tissue-specific based on its unique loop structure [7]. It has been reported that circRNA takes an essential function in myocardial infarction. For example, circMACF1 exacerbates the AMI through upregulating the expression of EMP1 and becoming a direct target of miR-500b-5p [8]. The finding proved that suppressing cardiomyocyte apoptosis and inflammation can mitigate AMI through knockdown of circ_0060745 [9]. Circular RNA CDR1 can treat arrhythmias in post-infarcted [10]. circCDYL can enhance cardiomyocyte proliferation by sponging miR-4793-5p [11]. circ_0091761 is located on the X chromosome and has a section length of 709 bp. The expression of circ_0091761 was dysregulated in the coronary arteries, which may be linked to the AMI process [12]. In addition, inhibition of circ_0091761 can protect the heart failure (HF) after myocardial infarction [13]. The research on the role of circ_0091761 in endothelial injury induced by AMI is relatively scarce and in need of further study.

MicroRNA (miRNA) is a class of small RNA that can participate in the post-transcriptional regulation of target genes [14]. It had reported that multiple miRNAs participated in the AMI processes. miR-133a-3p could play an active role in cardioprotection by mediating the protection of macrophage migration inhibitory factor engineered ucMSCs (MIF-Exo) [15]. The cardiac repair postinfarction was involved in miR-125a-5p through promoting macrophage M2 polarization [16]. miR-1278 also has been reported to be a target to protect the heart from myocardial infarction [14]. Nevertheless, the regulatory mechanism of miR-1278 in endothelial injury because of AMI remains indistinct.

This study focused on the level of circ_0091761 in AMI. Afterwards, the model of HUVECs was utilized to simulate an AMI model in order to elucidate the potential regulatory mechanisms between circ_0091761 and miR-1278 in endothelial injury caused by AMI.

## Materials and methods

### Ethical approval

The experimental procedures of this study were approved by the Ethics Committee of Zhangqiu District People’s Hospital and all subjects gave informed consent and signed an informed consent form.

### Patients and sample collection

A total of 45 patients with AMI were enrolled at Zhangqiu District People’s Hospital. And 52 healthy controls who underwent a medical test at the hospital during the same period were included in the control group. All patients had not received any treatment prior to blood sampling, and healthy controls had no cardiovascular disease history. “Fourth Universal definition of myocardial infarction” is the basis for diagnosis of AMI. All patients and healthy controls were required to collect fasting venous blood collected in the morning immediately after hospital admission. Venous blood was centrifuged at 2500rmp/min to obtain the upper serum layer, and stored at -80℃ for subsequent analyses.

### Cell culture

Human umbilical vein endothelial cells (HUVECs) were purchased from Bnbio. The control group were cultured in a humidified environment at 37℃, 5% CO_2_ using endothelia cell growth medium. The hypoxia group were submitted to hypoxia condition for 6, 12 and 24 h respectively, then cultured in normal condition.

### Cell transfection

Small interfering RNA against circ_0091761 (named as si-circ_0091761) and miR-1278 inhibitor, as well as their corresponding controls (si-NC, inhibitor NC) were obtained from GenePharma. The above plasmids or oligonucleotide fragments were used for cell transfection using Lipofectamine2000 reagent (Invitrogen, USA) in accordance with the given program.

### RNA extraction and Real-time quantitative reverse transcription PCR (RT-qPCR)

Total RNA was extracted from the serum and cells by TRIzol reagent (Invitrogen, USA) as the manufacture’s protocol. The quality and purity of the extracted RNA was measured using a spectrophotometer (260/280 nm ratio of 1.8 ~ 2.2). After RNase-R treatment, the cDNA was synthesized by reverse transcription of the tested RNA with TaqMan miRNA RT Kit. The specific expression levels were obtained by RT-qPCR on a 7300 Real-Time PCR system using the SYBR Green qPCR Master Mix. Finally, GAPDH and U6 were used as internal references for circRNAs and miRNAs, in addition to calculating Ct values using the 2-DeltaDeltaCt method. The levels of intercellular cell adhesion molecule-1 (ICAM-1) and vascular cell adhesion molecule-1 (VCAM-1) were also examined by RT-qPCR.

### Flow cytometry

Apoptosis was detected by flow cytometry utilizing the Annexin-FITC/propidium lodide (PI) kit (BD Biosciences, USA). Treated cells were trypsinized and then centrifuged to remove the supernatant. Cells were rinsed with PBS, suspended with 100 mul 1x buffer, and then cells were stained with AnnexinV/PI and incubated in the dark for 15 min at room temperature. Finally, the percentage of apoptosis was measured by flow cytometry.

### Measurement of intracellular reactive oxygen species content and lactate dehydrogenase

Intracellular reactive oxygen species (ROS) and lactate dehydrogenase (LDH) levels were measured by Oxiselect Intracellular ROS Assay Kit - Green Fluorescence (Cell Biolabs, USA) and LDH Assay Kit (Beyotime, China), respectively. The fluorescence intensity of ROS was detected using a Bio-Tek Fluorescence Microplate Reader and the LDH absorbance value was measured at 450 nm by a microplate reader.

### Subcellular localization

To determine the cellular localization of circ_0091761, subcellular separation of the cytoplasm and nucleus was achieved using the PARIS Kit (Invitrogen, USA), according to the manufacturer’s instructions. The levels of circ_0091761 in cytoplasm and nucleus were detected by RT-qPCR using GAPDH as cytoplasmic control and U6 as nuclear control, respectively.

### Bioinformatics analysis

The study utilized ENCORI and circular RNA Interactom databases to predict the target miRNA of circ_0091761, and use Venn diagrams to obtain overlapping target genes.

### Dual- luciferase reporter experiments

ENCORI predicts potential targets between miR-1278 and circ_0091761. The luciferase reporter vector circ_0091761-wt was constructed by inserting the wild-type sequence containing the miR-1278 target site circ_0091761 into the pGL3 vector. Also, circ_0091761-mut report vector was constructed by mutating the potential target site of miR-1278. The vectors were then co-transfected into HUVECs using Lipofectamine 2000. Luciferase activity was assayed with the Dual-Glo Luciferase Assay System kit (Promega, USA).

### RNA binding protein Immunoprecipitation assay

RNA-binding protein immunoprecipitation (RIP) assay was conducted with strict reference to Millipore kit (Millipore, USA) instructions. Cells were lysed with an equal volume of lysis buffer for 5 min, followed by centrifugation. The supernatant was incubated overnight at 4℃ with the detection antibody and then warmed to room temperature for 1 h. Afterwards, magnetic beads were added to obtain the complex, and RNA was obtained with buffer, and the content was detected using RT-qPCR.

### RNA pull-down assay

Biotin-labeled negative control (NC) and circ_0091761 mimic, designated as biotin-NC and biotin- circ_0091761, were used to incubate with streptavidin magnetic beads, and then both were incubated with cell lysates at 37℃ for 30 min, respectively. The RNA complexes pulled down by the magnetic beads were collected, and miR-1278 expression in the RNA complexes were qualified via RT-qPCR.

### Statistical analysis

All data analysis and plotting were performed using GraphPad Prism 9. Differences between two groups and differences between multiple groups were determined by Student’s t-test and one-way ANOVA, respectively. Correlations were calculated using Pearson’s correlation coefficient, with at least three independent replications of each experiment and *P* < 0.05 for statistically significant differences.

## Results

### circ_0091761 expression in patients with AMI and hypoxia-induced HUVECs

**Fig. 1:**
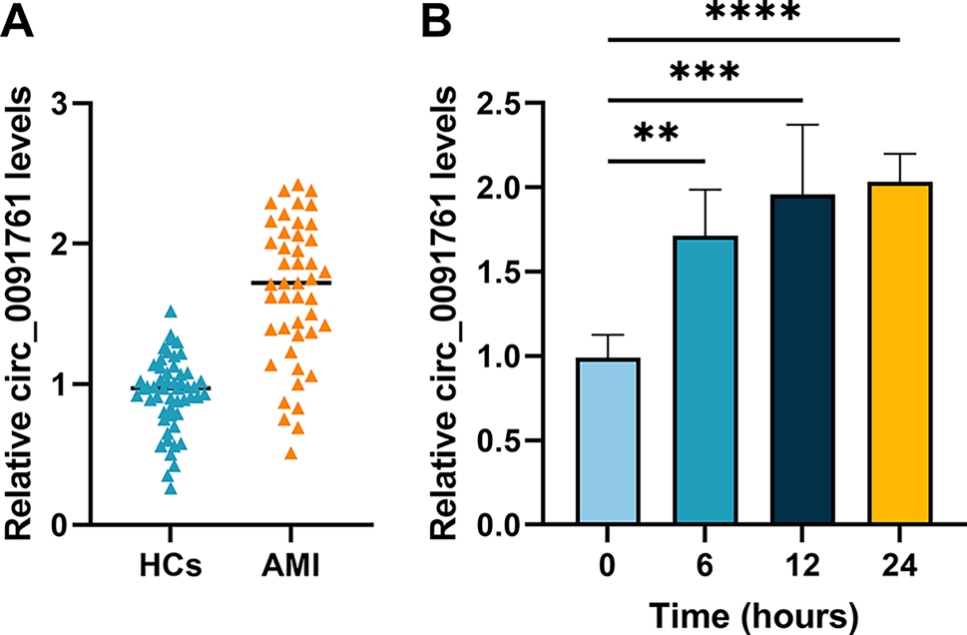
Expression of circ_0091761 in AMI and hypoxia-induced HUVECs. **A**. The level of circ_0091761 in AMI. **B**. The level of circ_0091761 expression in hypoxia-induced HUVECs at different times (0, 6, 12 and 24 h). **P < 0.01, ***P < 0.001, ****P < 0.0001 vs. 0 h

To investigate the role of circ_0091761 in AMI, 45 patients with AMI and 52 healthy controls were enrolled for this study. The level of circ_0091761 was significantly upregulated in AMI patients compared to healthy controls (*P* < 0.0001, Fig. 1A). Meanwhile, RT-qPCR analysis was used to detect the expression level of circ_0091761 in HUVECs at 0, 6, 12 and 24 h of hypoxia, respectively. The data indicated that circ_0091761 is significantly upregulated in hypoxia-induced HUVECs (*P* < 0.01, Fig. 1B).

### Knockdown of circ_0091761 attenuated hypoxia-induced cell injury in HUVECs

**Fig. 2:**
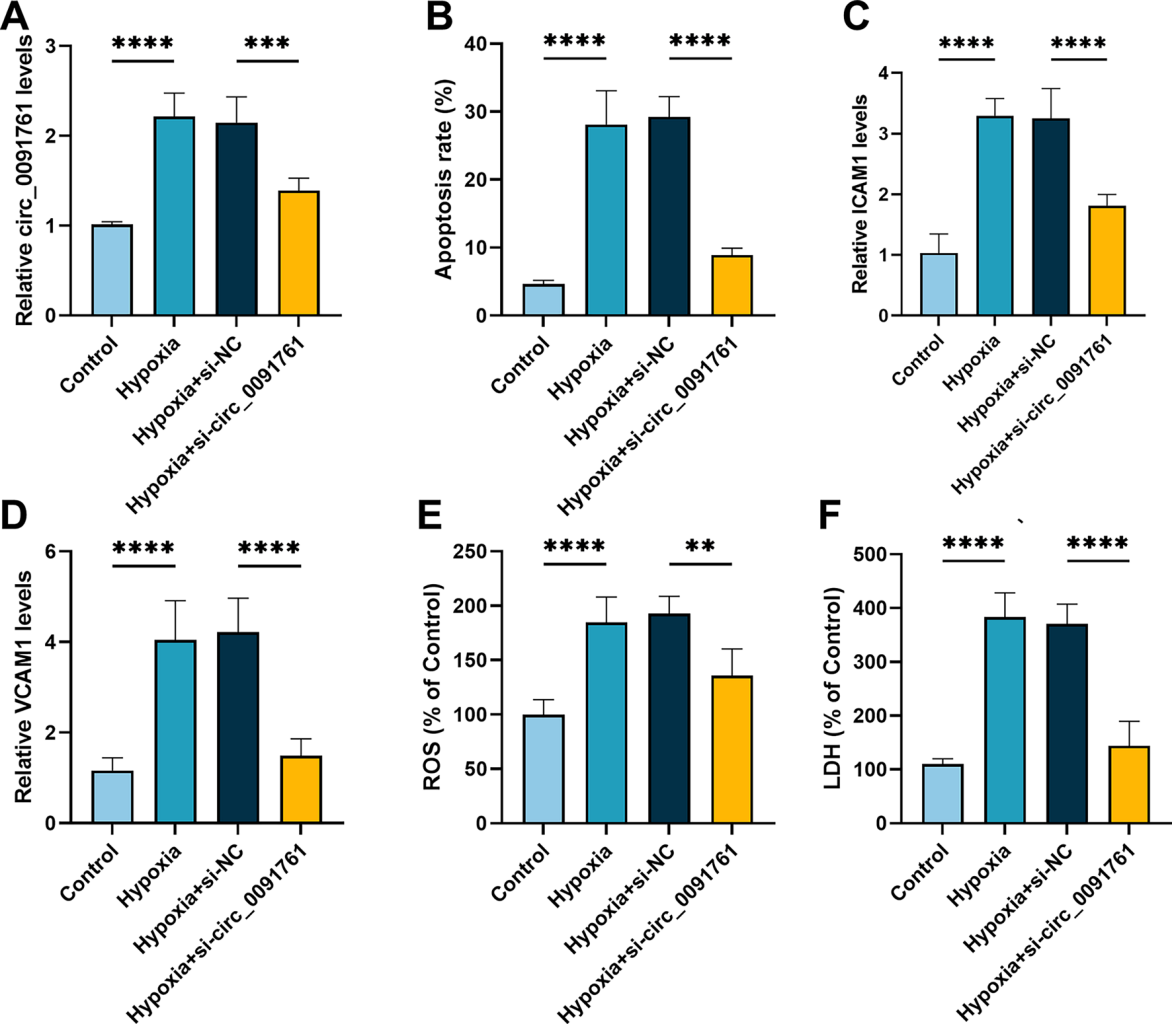
Silencing circ_0091761 attenuates hypoxia-induced cell injury in HUVECs. **A**. The level of circ_0091761 in HUVECs transfected with si-circ_0091761 and si-NC was assessed by RT-qPCR. **B**. Apoptosis rates of hypoxic and hypoxia + si-circ_0091761-treated HUVECs and their corresponding controls were examined by flow cytometry. **C-D.** The level of ICAM1 and VCAM1 were examined by RT-qPCR in hypoxic and hypoxia + si-circ_0091761-treated HUVECs together with their equivalent controls. **E** and **F**. ROS generation and LDH release were tested in control and treated HUVECs by using ROS assay kit and LDH assay kit. **P < 0.01, ***P < 0.001, ****P < 0.0001 vs. control or hypoxia + si-NC

To investigate the circ_0091761 function in hypoxia-induced cell injury, si-circ_0091761 and si-NC were transfected into HUVECs and the knockdown efficiency of circ_0091761 was confirmed (*P* < 0.001, Fig. 2A). Hypoxia-induced apoptosis in HUVECs cells can be significantly suppressed by silencing circ_0091761 (*P* < 0.0001, Fig. 2B). We tested the levels of pro-inflammatory molecules ICAM1 and VCAM1 utilizing RT-qPCR, which found that silencing circ_0091761 was able to dramatically reduce the levels of both (*P* < 0.0001, Fig. 2C-D). In addition, hypoxia-induced ROS production and LDH release in HUVECs could be significantly inhibited by knockdown of circ_0091761 (*P* < 0.01, Fig. 2E-F). Taken together, these findings indicate that hypoxia-induced cellular injury in HUVECs can be attenuated by down-regulating circ_0091761.

### Analysis of circ_0091761 target miRNAs

**Fig. 3:**
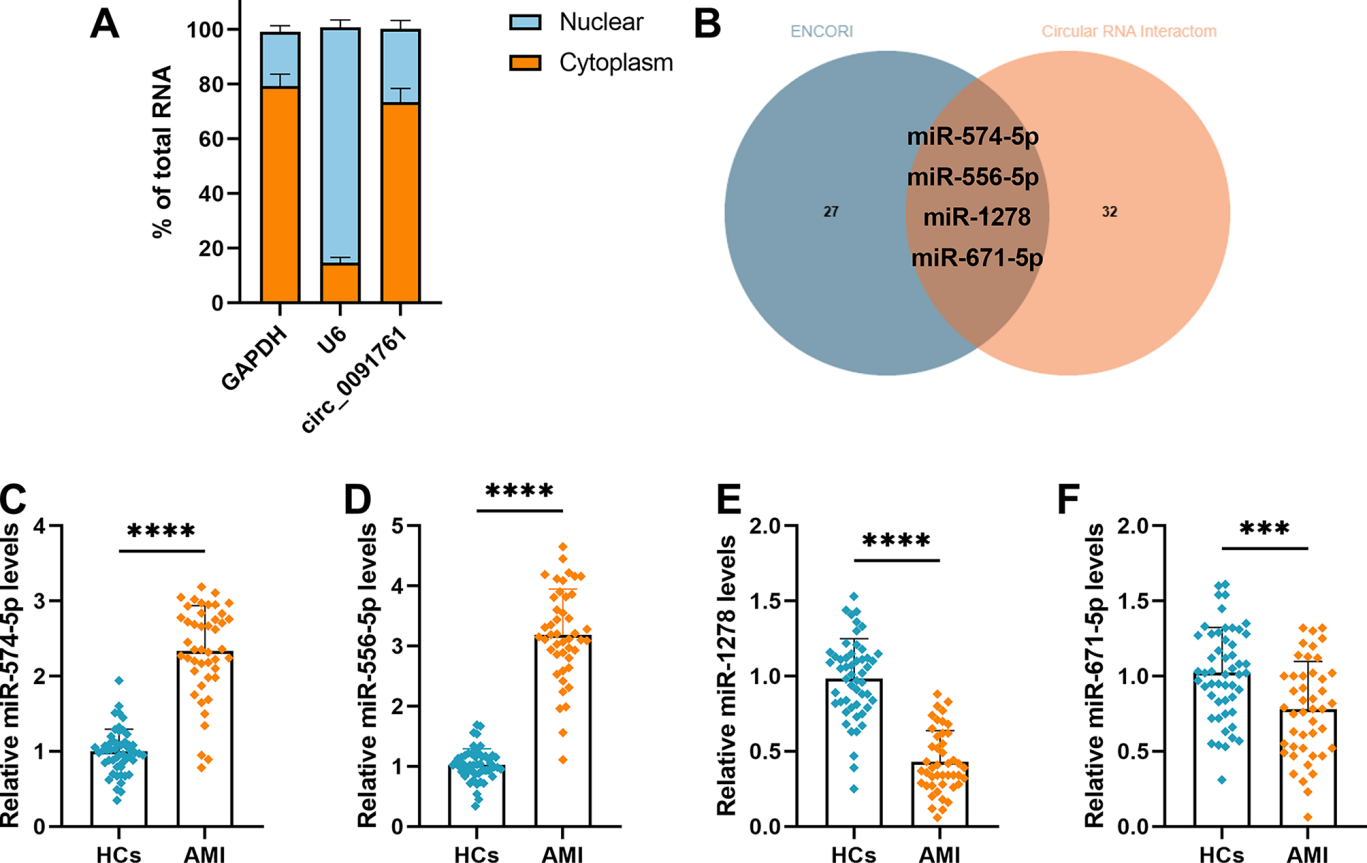
The analysis of circ_0091761 targets miRNA. **A**. Analysis of subcellular localization of circ_0091761. **B**. Venn diagram of circ_0091761 overlapping target miRNAs predicted from two databases. **C**-**F**. Levels of four target miRNAs (miR-574-5p, miR-556-5p, miR-1278, and miR-671-5p) were examined by RT-qPCR in patients with AMI (*n* = 45) as well as in health controls (*n* = 52). ****P < 0.0001 vs. HCs

Most of circ_0091761 was found to be localized in the cytoplasm (Fig. 3A). In order to investigate the role of the target miRNA for circ_0091761, the ENCORI and circular RNA Interactom databases predicted the target miRNA for circ_0091761, revealing that there were four miRNA (miR-547-5p, miR-556-5p, miR-1278 and miR-671-5p) duplicates in these two databases (Fig. 3B). AMI patients were significantly higher in miR-574-5p and miR-556-5p, while both miR-1278 and miR-671-5p were significantly lower compared to healthy controls, as analyzed by RT-qPCR (*P* < 0.001, Fig. 3C-F). Notably, miR-1278 had the lowest expression, so it was chosen for subsequent experiments.

### circ_0091761 targeted miR-1278 and negatively regulated miR-1278 expression

**Fig. 4:**
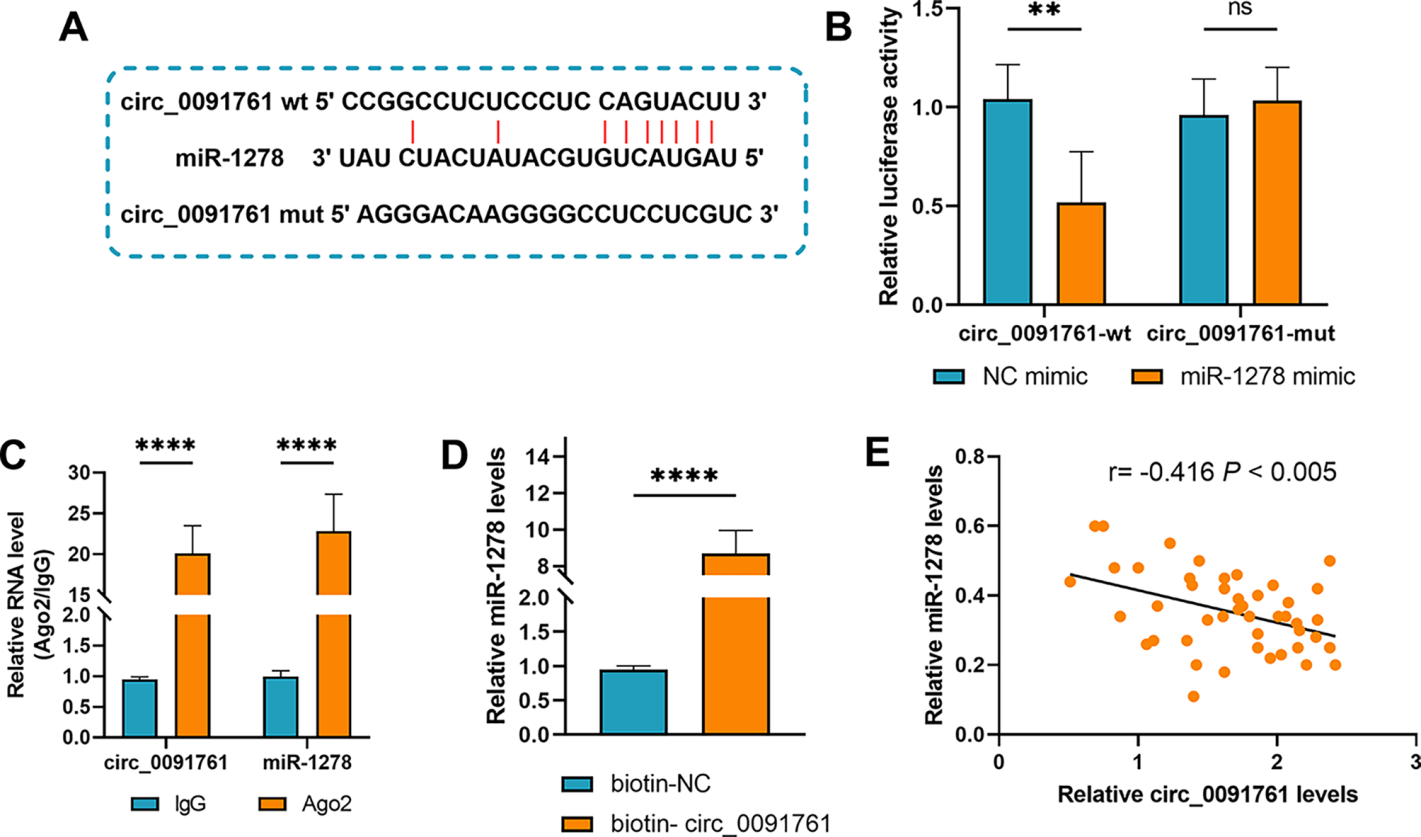
circ_0091761 targets miR-1278 that negatively regulates miR-1278 expression. **A**. Potential binding sites between circ_0091761 and miR-1278 were predicted with ENCORI. **B**-**D.** To verify the interaction between circ_0091761 and miR-1278, dual luciferase reporter assay, RIP assay and RNA pull-down assay were performed. **E**. The relationship between circ_0091761 and miR-1278 levels was analyzed by Pearson’s correlation coefficient. **P < 0.01, ****P < 0. 0001, *P* < 0.005

ENCORI was utilized to forecast potential binding sites for circ_0091761 and miR-1278 (Fig. 4A). miR-1278 significantly suppressed the luciferase activity of circ_0091761-wt, but not that of circ_0091761-mut, in HUVECs, as demonstrated by the dual-luciferase reporter gene assay (*P* < 0.01, Fig. 4B). Afterwards, it was noticed via RIP experiments that both circ_0091761 and miR-1278 were dramatically enriched in antibody Ago2, rather than antibody IgG (*P* < 0.0001, Fig. 4C). In the RNA pull-down assay, it was indicated that the miR-1278 contents were dramatically elevated in the biotin-circ_0091761 group versus biotin-NC group (*P* < 0.0001, Fig. 4D). As demonstrated in the outcome, circ_0091761 was negatively linked to miR-1278 (*r* = -0.416, *P* < 0.005) (*P* < 0.005, Fig. 4E). To summarize, our results suggest that circ_0091761 directly interacts with miR-1278 and negatively regulate miR-1278 expression.

### Inhibition of miR-1278 increased the effect of circ_0091761 silencing on hypoxia-induced cell injury in HUVECs

**Fig. 5:**
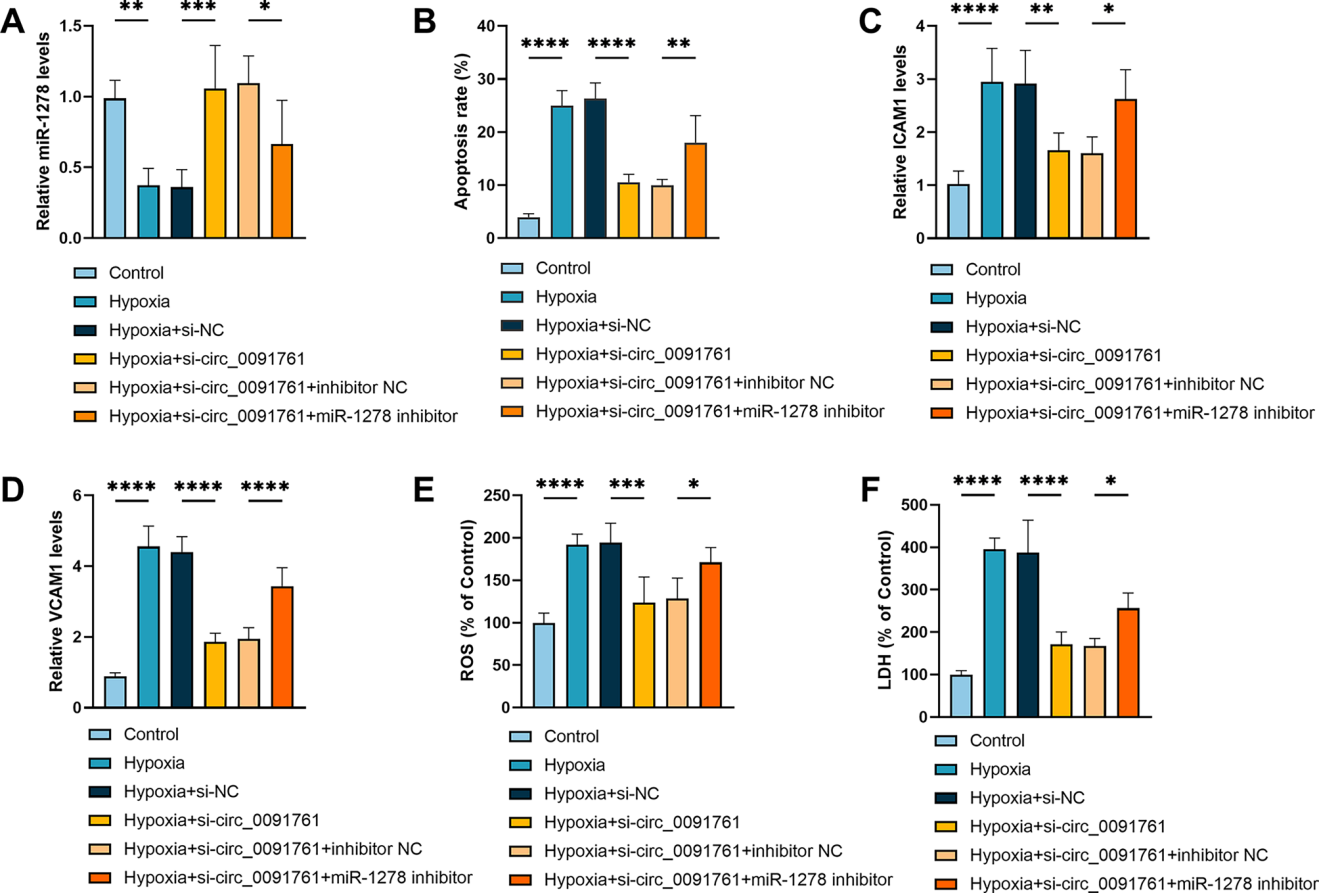
circ_0091761 modulates endothelial injury through targeting miR-1278. **A**. Levels of miR-1278 in HUVECs after transfection with si-circ_0091761 and miR-1278 inhibitor by RT-qPCR. **B**. Effect of transfection with si-circ_0091761 and miR-1278 inhibitor on apoptosis in HUVECs. **C**-**D.** The levels of si-circ_0091761 and miR-1278 inhibitor transfection on ICAM1 and VCAM1 in HUVECs. **E**-**F**. The rate of si-circ_0091761 and miR-1278 inhibitor transfection on ROS generation and LDH release in HUVECs. *P < 0.05, **P < 0.01, ***P < 0.001, ****P < 0.0001

To investigate the regulatory mechanisms of miR-1278 and circ_0091761 in hypoxia-induced cell injury, we co-transfected HUVECs with si-circ_0091761 in combination with miR-1278 inhibitor. It confirmed that silencing circ_0091761 markedly raised the expression level of miR-1278 in HUVECs and the knockdown efficiency of miR-1278 inhibitor (*P* < 0.05, Fig. 5A). Afterwards, we examined apoptosis in hypoxia-induced HUVECs transfected with si-circ_0091761 and si-circ_0091761 + miR-1278 as well as their corresponding controls, which showed that miR-1278 inhibitor inverted circ_0091761 silencing-mediated on hypoxia-induced HUVECs apoptosis (*P* < 0.01, Fig. 5B). Meanwhile, we found that miR-1278 inhibitor was able to invert silencing circ_0091761-mediated levels of ICAM1 and VCAM1 in hypoxia-induced HUVECs (*P* < 0.05, Fig. 5C-D). The trend of reduced LDH leakage rate and ROS content in the hypoxia + si-circ_0091761 group was reversed after additional transfection with miR-1278 inhibitor (*P* < 0.05, Fig. 5E-F). Collectively, circ_0091761 mediated hypoxia-induced cellular injury in HUVECs by sponging miR-1278.

## Discussion

Endothelial injury and thrombus formation are usually regarded as features of AMI. Vascular endothelial cells, which are located between the blood and the tissues, are physiologically characterized by injury that is generally manifested in the promotion of vasodilatation and resistance to aggregation [17, 18]. Endothelial cell injury and death are considered the initial events in endothelial pathology, and thus the integrity of the vascular endothelium acts as a vital factor in maintaining vascular homeostasis [19]. Hence, it is quite imperative to go for prevention and therapy of AMI by mitigating endothelial injury.

Multiple studies have implicated circRNAs as playing a regulatory role in endothelial injury and cardiovascular disorders. For instance, LPS-induced cerebral microvascular endothelial injury can be attenuated by circ_0057583 modulation of miR-204-5p/NR4A1 axis [20]. Up-regulation of TRIM14 acts on miR-330-5p via circIRAK1 to cause oxidized-LDL-induced endothelial cell injury in atherosclerosis [21]. Increasing the expression level of miR-1184 by knockdown of circROBO2 attenuates AMI [22]. Proliferation, cell cycle progression, and angiogenesis of HUVECs are modulated through the interaction of circEIF4G2 and MiR-26a, which is involved in influencing AMI [23]. CircMACF1 alleviates myocardial fibrosis after acute myocardial infarction through miR-16-5p/SMAD7 signaling pathway as a novel target for AMI treatment [24]. circ_0091761 has also been reported to be capable of impacting acute myocardial infarction, but the underlying physiologic mechanism is unclear. In the present research, we examined the levels of circ_0091761 in patients with AMI and controls, as well as in HUVECs induced by hypoxia for different durations using RT-qPCR, which found that circ_0091761 was markedly up-regulated in AMI patients, and the up-regulation was more pronounced with the longer time of hypoxia induction in HUVECs. As cell adhesion molecules, high expression of VCAM1 and ICAM1 can promote the adhesion of white blood cells to endothelial cells, exacerbate inflammatory responses and vascular endothelial permeability, and serve as key markers of endothelial injury [25]. Excessive ROS can trigger oxidative stress, damage the DNA, lipids, and proteins of endothelial cells, and lead to cell apoptosis and dysfunction [26]. As an indicator of cytotoxicity, an increase in the release of LDH reflects the disruption of cell membrane integrity and serves as direct evidence of endothelial cell death [27]. Following this, we investigated the function of circ_0091761 in hypoxia-induced cell injury, which revealed that silencing circ_0091761 significantly diminished apoptosis rate and the levels of ICAM1 and VCAM1. Meanwhile, the generation of ROS and the release of LDH were also significantly reduced in hypoxia + si-circ_0091761-treated HUVECs. It is evident that down-regulation of circ_0091761 attenuates AMI-induced endothelial cell injury.

circRNAs can indirectly modulate gene expression via acting as molecular sponges for miRNAs. circNMD3 inhibits x-LDL-induced injury to HUVECs as a molecular sponge for miR-498 [28]. circ_0090231 can be involved in oxidized-LDL-mediated injury to HUVECs through the miR-9-5p/TXNIP axis [29]. In order to explore the target miRNA of circ_0091761, we analyzed it using bioinformatics and realized that miR-1278 is probably the target of circ_0091761. miR-1278 is known to be involved in the development of gastric cancer and glioma [30, 31]. miR-1278 is a target that mediates inflammation and apoptosis in cardiomyocytes [14]. Through RT-qPCR detection of the expression levels of four candidate miRNAs in AMI patients and healthy controls, miR-1278 was found to have the most significant downregulation in AMI patients (*P* < 0.0001), and its expression level was the lowest among the four miRNAs (Fig. 3C-F). Combining bioinformatics prediction (intersection of two databases) and literature reports [14], it is speculated that it has the strongest correlation with endothelial injury regulation. Therefore, miR-1278 was prioritized as the target for subsequent mechanistic studies. To probe further whether there is indeed target binding between circ_0091761 and miR-1278. We verified that miR-1278 is indeed a target of circ_0091761 and is negatively regulated by circ_0091761 in hypoxia-induced HUVECs with dual-luciferase reporter assay, RIP assay and RNA pull-down assay. In addition, clinical sample analysis showed a significant negative correlation between circ_0091761 and miR-1278 (*r* = -0.416, *P* < 0.005), which further supports the interaction between the two. This correlation suggests that during the pathological process of AMI, the high expression of circ_0091761 may sponge miR-1278, reducing its expression level, thereby relieving the inhibition of downstream pro-injury pathways and ultimately exacerbating endothelial injury. This clinical-level association echoes the in vitro mechanistic studies, providing more comprehensive evidence for the regulatory role of the circ_0091761/miR-1278 axis in AMI-induced endothelial injury. Experimentally we demonstrated that in hypoxia-induced HUVECs, miR-1278 inhibition reverted the circ_0091761 silencing-mediated inhibition of apoptosis, ICAM1 and VCAM1 levels. At the same time, miR-1278 inhibition also rescued the ROS level and LDH release. To summarize, our findings demonstrate that circ_0091761 mediates endothelial injury induced by AMI through targeting miR-1278.

Notably, this study has certain limitations: it only explored the role of the circ_0091761/miR-1278 axis through an in vitro cell model (hypoxia-induced HUVECs), lacking animal experiments to verify the biological function of this regulatory axis in vivo. Moreover, the clinical sample size is relatively limited (45 AMI patients), which may affect the generalizability of the results. In addition, the downstream target genes of miR-1278 and the specific signaling pathways they mediate have not been deeply explored, and the analysis of the molecular mechanism of endothelial injury still needs to be improved. Future research will focus on the following directions: constructing an AMI animal model (such as a rat coronary artery ligation model) to verify the expression changes and intervention effects of circ_0091761 in vivo; expanding the clinical sample size and analyzing the diagnostic value of circ_0091761 in combination with patients’ clinical characteristics (such as infarct size, prognostic score); screening the downstream target genes of miR-1278 through gene chip or sequencing technology to clarify the complete mechanism of the “circ_0091761/miR-1278/target gene” axis in regulating endothelial injury. In addition, the clinical transformation potential of circ_0091761 is worthy of attention: its high expression in the serum of AMI patients and its correlation with the disease process make it expected to be developed as a non-invasive serum marker for the early diagnosis of AMI; small interfering RNA (siRNA) or antisense oligonucleotide (ASO) targeting circ_0091761 can reduce endothelial injury by inhibiting its expression, providing a new strategy for the targeted therapy of AMI. Subsequent studies need to verify its in vivo safety and effectiveness through animal experiments, and gradually advance to preclinical research to explore the feasibility of its translational application.

On the basis of our data, we demonstrated that inhibition of circ_0091761 ameliorated endothelial injury induced by AMI by regulating miR-1278, which provides in vitro evidence for circ_0091761 as a potential therapeutic target in AMI.

## Data Availability

The datasets used and/or analysed during the current study are available from the corresponding author on reasonable request.
